# Role of *Saccharomyces* Single-Stranded DNA-Binding Protein RPA in the Strand Invasion Step of Double-Strand Break Repair

**DOI:** 10.1371/journal.pbio.0020021

**Published:** 2004-01-20

**Authors:** Xuan Wang, James E Haber

**Affiliations:** **1**Rosenstiel Center and Department of Biology, Brandeis UniversityWaltham, MassachusettsUnited States of America

## Abstract

The single-stranded DNA (ssDNA)-binding protein replication protein A (RPA) is essential for both DNA replication and recombination. Chromatin immunoprecipitation techniques were used to visualize the kinetics and extent of RPA binding following induction of a double-strand break (DSB) and during its repair by homologous recombination in yeast. RPA assembles at the HO endonuclease-cut *MAT* locus simultaneously with the appearance of the DSB, and binding spreads away from the DSB as 5′ to 3′ exonuclease activity creates more ssDNA. RPA binding precedes binding of the Rad51 recombination protein. The extent of RPA binding is greater when Rad51 is absent, supporting the idea that Rad51 displaces RPA from ssDNA. RPA plays an important role during *RAD51*-mediated strand invasion of the *MAT* ssDNA into the donor sequence *HML*. The replication-proficient but recombination-defective *rfa1-t11* (K45E) mutation in the large subunit of RPA is normal in facilitating Rad51 filament formation on ssDNA, but is unable to achieve synapsis between *MAT* and *HML*. Thus, RPA appears to play a role in strand invasion as well as in facilitating Rad51 binding to ssDNA, possibly by stabilizing the displaced ssDNA.

## Abstract 2

By studying the repair of double-strand DNA breaks in vivo, evidence of a new role for the DNA-binding protein RPA has been discovered

## Introduction

Repair of double-strand breaks (DSBs) by homologous recombination involves the search for homology to locate an intact donor sequence. The search is successful when the broken DNA molecule basepairs with the homologous template, termed synapsis, and forms strand invasion intermediates of recombination. In budding yeast and other higher eukaryotes, this process requires both the Rad51 strand exchange protein and the single-stranded DNA (ssDNA)-binding protein replication protein A (RPA) (Alani et al. 1992; Shinohara et al. 1992; Ogawa et al. 1993; Sung 1994; Symington 2002). RPA was first discovered through its essential role in SV40 DNA replication in vitro as a ssDNA-binding protein (Wold et al. 1989). The RPA complex forms a heterotrimer, which consists of three subunits of 70, 34, and 14 kDa, encoded by the *RFA1*, *RFA2*, and *RFA3* genes, respectively (Wold 1997). Deletion of any of these genes leads to lethality in yeast (Heyer et al. 1990; Brill and Stillman 1991). The biological function of RPA was further demonstrated to be important in homologous recombination in Saccharomyces cerevisiae (Alani et al. 1992; Firmenich et al. 1995; Umezu et al. 1998) and in other aspects of DNA metabolism. Cells carrying a point mutation (K45E) in the largest subunit of RPA (*rfa1-t11*) are proficient for DNA replication, but their ability to perform mating-type (*MAT*) gene switching, single-strand annealing, and meiotic recombination is severely impaired (Umezu et al. 1998; Soustelle et al. 2002).

Because RPA is essential for DNA replication, a great deal about its role in recombination has been learned from in vitro studies of Rad51-mediated strand exchange (Bianco et al. 1998; Symington 2002). These studies have shown that RPA facilitates the formation of continuous Rad51 filaments on ssDNA by removing inhibitory secondary structures (Alani et al. 1992; Sugiyama et al. 1997, 1998). A similar requirement is seen in bacteria, where the ssDNA-binding protein SSB apparently plays an analogous role to allow the Rad51 homologue RecA to polymerize across regions that contain secondary structures (Shibata et al. 1980; West et al. 1981; Kowalczykowski and Krupp 1987; Kuzminov 1999). Rad51 further displaces RPA, while RecA displaces SSB, leading to the filament that facilitates the search for homologous double-stranded DNA (dsDNA) sequences and then catalyzes strand invasion and the formation of a displaced single strand (Kowalczykowski et al. 1987; New et al. 1998; Eggler et al. 2002; Sugiyama and Kowalczykowski 2002). However, order-of-addition experiments have suggested that if RPA/SSB is added to ssDNA prior to Rad51/RecA, successful displacement will not occur because RPA/SSB has higher affinity for ssDNA, unless mediator proteins, such as Rad52 and Rad55/Rad57 in yeast and RecO/RecR in bacteria, are present (Umezu et al. 1993; New et al. 1998; Shinohara and Ogawa 1998; Kuzminov 1999; Sugiyama and Kowalczykowski 2002; Symington 2002). But if Rad51/RecA has polymerized onto ssDNA first under conditions that prevent the formation of secondary structures, further addition of RPA/SSB will stimulate in vitro strand exchange in a species-specific manner (Heyer and Kolodner 1989; Morrical and Cox 1990; Sung 1994; Sugiyama et al. 1997).

Using the same in vitro system, Kantake et al. (2003) have examined the effects of the *rfa1-t11* (Rfa1-K45E) mutation on strand exchange with Rad51. Although *rfa1-t11* protein bound to ssDNA identically to wild-type and could stimulate strand exchange if Rad51 was preloaded onto ssDNA, the mutant protein exhibited delayed and less-efficient strand exchange if it was first bound to ssDNA, especially at higher concentrations, even in the presence of Rad52. This defect was explained by the slow displacement of *rfa1-t11* from ssDNA by Rad51.

Recently, immunostaining experiments have been carried out in S. cerevisiae as well as in higher eukaryotes to investigate the association of RPA to DSBs following γ-irradiation and during meiosis. These studies have suggested that RPA and Rad51 form subnuclear foci at sites of ssDNA after irradiation and during meiotic recombination (Gasior et al. 1998; Golub et al. 1998; Raderschall et al. 1999) and that RPA is recruited to these sites prior to Rad51 (Golub et al. 1998; Gasior et al. 2001).

In order to understand better how RPA is involved in DSB repair in vivo, we have looked at its function in *MAT* switching in yeast, which is the well-studied example of DSB-induced homologous recombination (Haber 2002a). *MAT* switching is initiated when HO endonuclease creates a site-specific DSB at the *MAT* locus, which is then repaired by gene conversion using one of the two heterochromatic donor sequences, *HML* or *HMR* (Pâques and Haber 1999; Haber 2000, Haber 2002a). By using a galactose-inducible HO endonuclease gene (Jensen et al. 1983), the induction of the DSB and its repair occur synchronously in a population of cells so that the kinetics of DSB repair and the appearance of intermediates of recombination can be followed by physical monitoring of the process via Southern blot and PCR assays (Haber 1995, Haber 2002a, Haber 2002b).

To learn more precisely about how RPA participates in homologous recombination in vivo, we have used chromatin immunoprecipitation (ChIP) assays (Dedon et al. 1991; Sugawara et al. 2003) to analyze the association of RPA and Rad51 to DNA as it undergoes *MAT* switching. At the same time, the fate of recombining DNA was analyzed by Southern blot and PCR techniques (White and Haber 1990; Haber 1995). The combination of these approaches has enabled us to visualize the kinetics and extent of RPA binding to a DSB and the homologous template during recombination. We report that the biological function of RPA is also required during the strand invasion step of recombination. *Rfa1-t11* mutant cells are not defective in Rad51 nucleoprotein filament assembly, as observed by ChIP, but are incapable of performing the strand exchange and thus the completion of DSB repair.

## Results

### Kinetics and Extent of RPA Binding at DSB Ends in the Absence of DNA Repair

In wild-type yeast cells, a DSB created at the *MAT* locus can be repaired by gene conversion with one of the two donor sequences, *HML* or *HMR*, or the DSB can be left unrepaired in most cells by deleting these donors (Haber 2002a). In order to monitor RPA binding to DSB ends, we first performed ChIP analysis on strains in which both of the donor loci were deleted so that the DSB at *MAT* could not be repaired and 5′ to 3′ exonuclease activity would generate resected ssDNA unimpeded for many hours (Lee et al. 1998). In these strains, nearly complete cutting of *MAT* by the galactose-induced HO endonuclease occurred within 20 min after induction (see below).

**Figure 1:**
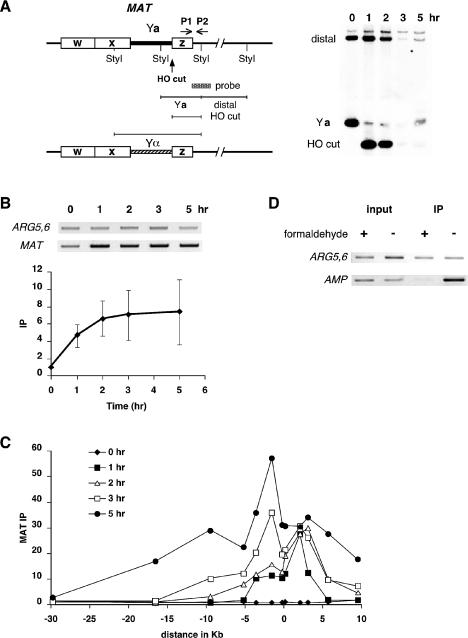
Recruitment of RPA to a DSB in the Absence of DNA Repair A strain deleted for donors (yXW1), thus incapable of repairing a DSB by gene conversion, was pregrown in YP–lactate medium, and 2% galactose was added to the culture to induce a DSB at *MAT*. DNA was extracted at intervals after HO cutting, to which polyclonal antibody against Rfa1 was applied to immunoprecipitate RPA-bound chromatin. Another set of DNA samples were taken at the same time for Southern blot analysis. (A) Map of *MAT* showing the locations of the HO-cut site as well as the StyI restriction sites and the primers (P1 and P2), 189 bp to 483 bp distal to the DSB, used to PCR-amplify RPA-associated *MAT* DNA from the immunoprecipitated extract. Purified genomic DNA was digested with StyI, separated on a 1.4% native gel, and probed with a ^32^P-labeled *MAT* distal fragment to monitor the appearance of the HO-cut fragment (see Materials and Methods). The 1-h timepoint represents 1 h after galactose induction of the HO endonuclease. (B) PCR-amplified RPA-bound *MAT* DNA in a wild-type strain (yXW1). As controls, primers to an independent locus, *ARG5,6* (see Materials and Methods), were used to amplify DNA from the immunoprecipitated chromatin. PCR samples were run on ethidium bromide-stained gels (reverse images are shown). Quantitated signals were graphed for the wild-type strain. IP represents ratio of the *MAT* IP signal to *ARG5,6* IP signal. Error bars show one standard deviation. (C) RPA-bound chromatin was PCR-amplified from sites located proximal and distal to the DSB and then quantitated and graphed as described in (B). The DSB is shown at 0 bp. (D) Effect of formaldehyde cross-linking on RPA binding to ssDNA. In both the noncross-linked samples and the cross-linked samples, 4 ng of single-stranded heterologous β-lactamase (*AMP*) gene DNA was added during the extract preparation step of ChIP. The amount of input genomic and heterologous DNA was measured by PCR primers specific to the *ARG5,6* locus and to the *AMP* sequence, respectively. RPA-associated *ARG5,6* and *AMP* DNA were analyzed from the IP samples. PCR samples were run on ethidium bromide-stained gels (reverse images are shown).

**Figure 2:**
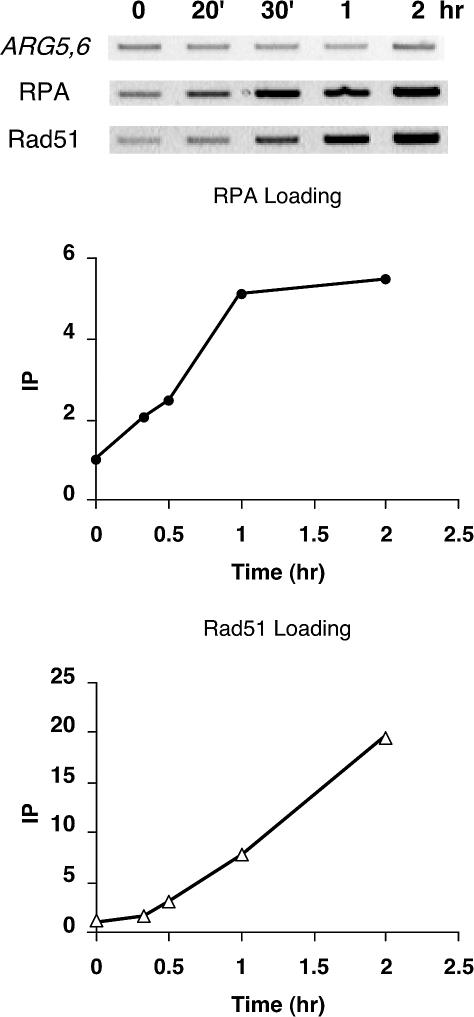
Timing of Recruitment of RPA versus Rad51 to the DSB An unrepairable DSB was created in the wild-type strain (yXW1), and closer timepoints were harvested at 20 min and 30 min after the HO cut. DNA samples extracted at each timepoint were split. One half was applied with antibody against Rfa1 to immunoprecipitate RPA-associated DNA, while the other half was applied with anti-Rad51 antibody to immunoprecipitate Rad51-bound chromatin. RPA- or Rad51-associated *MAT* DNA was PCR-amplified and run on ethidium bromide-stained gels (reverse images are shown). DNA signals were quantitated and graphed as described in Figure 1 for RPA ChIP. PCR-amplified *ARG5,6* signals from the input DNA were used as controls for quantitation and graphing for Rad51 ChIP (see Materials and Methods).

In wild-type cells, after HO induction, significant RPA binding to sequences close to the HO cleavage site was seen by ChIP (Figure 1A and 1B), using a pair of primers (P1 and P2) that amplify sequences 189 bp to 483 bp distal to the HO cut (Figure 1A). As shown in Figure 2, RPA was recruited to DSB ends as soon as the DSB could be detected on a Southern blot (20 min after induction). The binding of RPA increased for about 2–3 h, until presumably all sequences near *MAT* had been rendered single-stranded (Frank-Vaillant and Marcand 2002) (see Figure 1B). At later times, one detects RPA binding at increasing distances from the cleavage site, as these regions were rendered single-stranded by 5′ to 3′ exonuclease activity (Lee et al. 1998) (see Figure 1C).

In carrying out the ChIP measurements, we were aware that RPA is both abundant within the cell and binds strongly and cooperatively to ssDNA in vitro (Heyer and Kolodner 1989). It was possible that some of the RPA binding we measured by ChIP could have arisen after the cells were broken and could be independent of formaldehyde cross-linking. Indeed, in the absence of cross-linking, we found that there was substantial binding of RPA to the HO-cut *MAT* locus, which was resistant to both addition of 2 mg of ssDNA (equivalent to a 1,000-fold genome excess) at the time of cell breakage and washing with 4.7 M NaCl, although it greatly reduced background binding (data not shown). However, in formaldehyde cross-linked samples, there was no such adventitious binding of RPA to ssDNA regions, apparently because the formaldehyde-treated proteins are no longer able to bind. This was shown directly by adding 4 ng of purified single-stranded β-lactamase (*AMP*) gene DNA from plasmid pBR322 at the time of cell breakage. Whereas there was substantial ChIP of the *AMP* sequences in noncross-linked samples, there was almost no signal in cells that had first been treated with formaldehyde (see Figure 1D).

### RPA Binding Precedes the Binding of the Strand Exchange Protein Rad51

In vitro studies of the early steps of recombination have suggested that in order to make a continuous and functional nucleoprotein filament, RPA must bind before Rad51 to ssDNA to remove inhibitory secondary structures (Sugiyama et al. 1997). Indirect immunofluorescence experiments in S. cerevisiae have also suggested that RPA assembles before Rad51 at DSBs after γ-irradiation (Gasior et al. 2001). Therefore, the timing of recruitment of both RPA and Rad51 proteins to a DSB in vivo were compared by ChIP.

**Figure 4:**
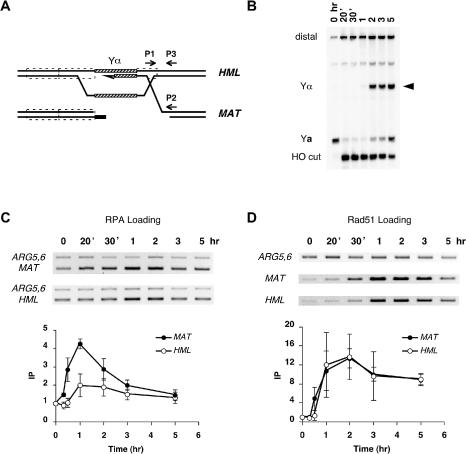
Localization of RPA and Rad51 to *HML* and *MAT* during DSB-Induced Gene Conversion A strain carrying an *HML*α donor (yXW2), thus able to repair the DSB at *MAT* by gene conversion, was treated with 2% galactose to induce HO endonuclease and then with 2% glucose after 1 h to repress further HO expression. DNA extracted at intervals after HO cutting was split. One half was applied with antibody against Rfa1 to immunoprecipitate RPA-associated DNA, while the other half was applied with anti-Rad51 antibody to immunoprecipitate Rad51-bound chromatin. Another set of DNA samples were taken at the same time for Southern blot analysis. (A) Diagram of *MAT* and *HML* showing the locations of the primers, 189 bp to 483 bp distal to the DSB at *MAT* (P1 and P2) and 189 bp to 467 bp from the uncleaved HO recognition site at *HML* (P1 and P3), used to PCR-amplify RPA- and Rad51-associated *MAT* and *HML* DNA from the immunoprecipitated extract. (B) Purified genomic DNA was digested with StyI, separated on a 1.4% native gel, and probed with a ^32^P-labeled *MAT* distal fragment to monitor the appearance of the HO-cut fragment and the repaired product Yα (see Figure 1A; see Materials and Methods). The arrowhead indicates the switched product Yα. RPA- and Rad51-bound *MAT* and *HML* DNA were PCR-amplified with primers P1 and P2 and with P1 and P3, respectively. Samples were run on ethidium bromide-stained gels. (C and D) Reverse images are shown for RPA ChIP (C) and Rad51 ChIP (D). DNA signals were quantitated and graphed as described in Figure 2. Error bars show one standard deviation.

RPA was detected at *MAT* 20 min after the HO cut, while Rad51 binding was not observed until the 30 min timepoint (Figure 2). Similar results were obtained in strains that are able to carry out gene conversion (see Figure 4). These observations strongly support the idea that RPA binding to HO-cut DNA precedes that of Rad51.

### In Vivo Competition between RPA and Rad51 for ssDNA

**Figure 3:**
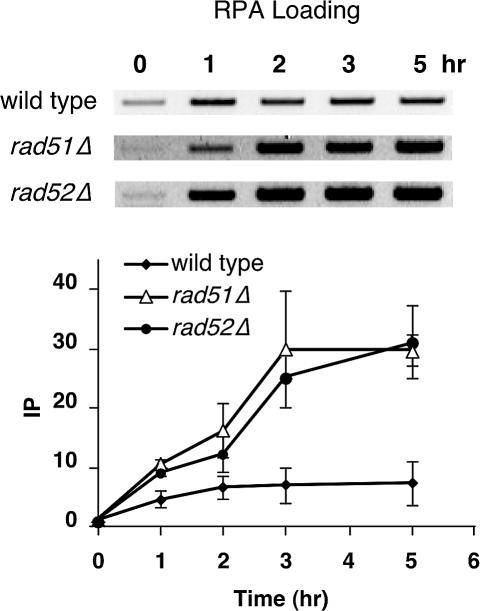
Effect of *rad51*Δ and *rad52*Δ on the Extent of RPA Binding to an Unrepairable DSB An unrepairable DSB was created in wild-type (yXW1), *rad51*Δ (ySL306), and *rad52*Δ (ySL177) strains and RPA-bound chromatin was immunoprecipitated using anti-Rfa1 antibody. PCR-amplified DNA from the *MAT* locus was run on ethidium bromide-stained gels (reverse images are shown). DNA signals were quantitated and graphed as described in Figure 1. Error bars show one standard deviation.

Studies of RPA in vitro would suggest that the amount of RPA bound to ssDNA may be limited by its displacement by Rad51, through the help of Rad52, and the Rad55/57 heterodimer (Sung 1997a, 1997b; New et al. 1998; Shinohara and Ogawa 1998; Sugiyama and Kowalczykowski 2002). To test this idea, we deleted *RAD51* and measured RPA binding at *MAT*. The extent of RPA binding was approximately 5- to 6-fold higher in the *rad51*Δ strain, consistent with this expectation (Figure 3). A similar result was found in a *rad52*Δ strain (Figure 3), supporting the hypothesis that the displacement of RPA by Rad51 depends on Rad52, which acts as a mediator between these two ssDNA-binding proteins (Sung 1997a; New et al. 1998; Song and Sung 2000; Sugiyama and Kowalczykowski 2002; Sugawara et al. 2003).

### RPA Is Recruited to Both the Donor and the Recipient Sequences during Gene Conversion

We then examined RPA in a strain in which the DSB at *MAT* **a** could be repaired by gene conversion, using *HML*α as the donor (Figure 4A). As soon as the DSB was visible, an increase in RPA binding was seen (Figure 4B and 4C). RPA binding increased for about 1 h and then decreased nearly to the baseline level about the time that *MAT* switching was completed (Figure 4B and 4C).

Importantly, RPA also appeared to become associated with the donor locus. This was detected by ChIP using the same primer P1 located in the *Z* region shared by *MAT* and *HML* and an *HML* sequence-specific primer P3 (Figure 4A). Whereas RPA could be found associated with *MAT* 20 min after HO induction, its association with *HML* was seen only after 1 h (Figure 4C). The association of RPA with *HML* came at about the same time as we saw synapsis between *HML* and *MAT* as revealed by ChIP with anti-Rad51 antibody (Figure 4D; also Sugawara et al. 2003). The extent of RPA binding to *HML* was substantially less than seen at *MAT* (Figure 4C), where ssDNA may extend further than the 320 bp of homology between *MAT* and *HML*; these more distal ssDNA sequences would not be involved directly in recombination. The lower amount of RPA binding at the donor locus may also arise from a lower concentration of RPA that is needed at the sites of strand invasion or a transient presence of RPA at those loci. But the fact that cross-linked RPA can immunoprecipitate the donor locus might indicate that RPA is recruited onto the single-stranded D-loop that is created by strand invasion. This would be consistent with in vitro studies of Rad51-mediated strand exchange that suggest that strand invasion per se can occur without RPA, but that the heteroduplex DNA is unstable unless the displaced strand is bound by RPA (Eggler et al. 2002). A similar requirement for SSB was suggested in RecA-mediated strand invasion (Lavery and Kowalczykowski 1992). We cannot entirely rule out the possibility that the apparent association of RPA with *HML* resulted from the cross-linking of synapsed *MAT* and *HML* sequences directly or through Rad51-containing cross-links and where RPA was bound to ssDNA sequences distal to the 320-bp homology shared by *MAT* and its donor. Further evidence of a role for RPA in synapsis will be presented below.

To understand better the dynamics between RPA and Rad51 during gene conversion, we also examined Rad51 recruitment to *MAT* and *HML* relative to that of RPA (Figure 4D). Rad51 was only detected at *MAT* 30 min postinduction (compared to 20 min for RPA). Rad51 binding increased for about 1 h and then remained bound for several hours. As reported previously (Sugawara et al. 2003), Rad51 showed a delayed association with the donor *HML*, reflecting the time required to form a functional filament and to search the genome for homologous sequences. These observations provide evidence of the time at which synapsis between *MAT* and *HML* is achieved. Here, too, Rad51 association with the donor remained for several hours, beyond the time when *MAT* switching is completed.

### *rfa1-t11* Mutant Cells Are Defective in the Synapsis Step of Gene Conversion

**Figure 6:**
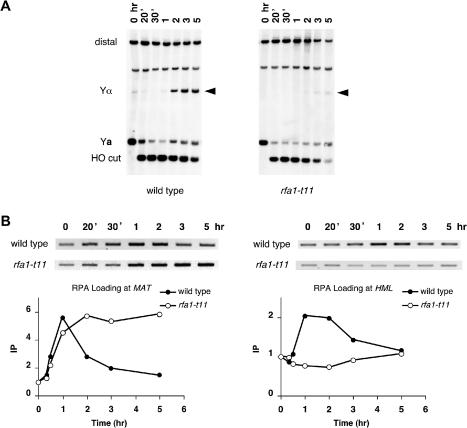
*rfa1-t11* Was Not Able to Associate with the Donor Sequence during Gene Conversion The wild-type strain carrying the *HML*α donor (yXW2) and an isogenic strain carrying the *rfa1-t11* mutation (yXW3) were treated with 2% galactose to induce HO endonuclease and then with 2% glucose after 1 h to repress further HO expression. DNA extracted at intervals after HO cutting was split. One half was applied with antibody against Rfa1 to immunoprecipitate RPA-associated DNA, while the other half was applied with anti-Rad51 antibody to immunoprecipitate Rad51-bound chromatin. Another set of DNA samples was taken at the same time for Southern blot analysis. (A) Purified genomic DNA was digested with StyI, separated on a 1.4% native gel, and probed with a ^32^P-labeled *MAT* distal fragment to monitor the appearance of the HO-cut fragment and the repaired product Yα (see Figure 1A; see Materials and Methods). Arrowheads indicate the switched product Yα. (B) RPA-bound *MAT* and *HML* DNA was PCR-amplified with primers P1 and P2 and with P1 and P3, respectively (see Figure 4A). Samples were run on ethidium bromide-stained gels (reverse images are shown). DNA signals were quantitated and graphed as described in Figure 1.

To learn more about RPA function during recombination, we investigated the behavior of the *rfa1-t11* (K45E) mutation in the largest subunit of RPA. This mutation has little effect on DNA replication per se, but severely impairs both gene conversion (*MAT* switching) and single-strand annealing pathways of homologous recombination (Umezu et al. 1998) (also Figure 6A). Cells containing this mutation displayed hyperresection at meiotic DSB ends and defects in the repair of these DSBs (Soustelle et al. 2002). In vitro biochemical studies have shown that *rfa1-t11* is displaced from ssDNA by Rad51 more slowly than wild-type RPA, and as a consequence, Rad51-mediated strand exchange is inhibited when the ssDNA is complexed with the mutant RPA heterotrimer (Kantake et al. 2003). Here, we examined binding of Rfa1-K45E in vivo by ChIP and also its effect on Rad51 localization to an HO-induced DSB, using the same antibodies as against wild-type RPA.

**Figure 5:**
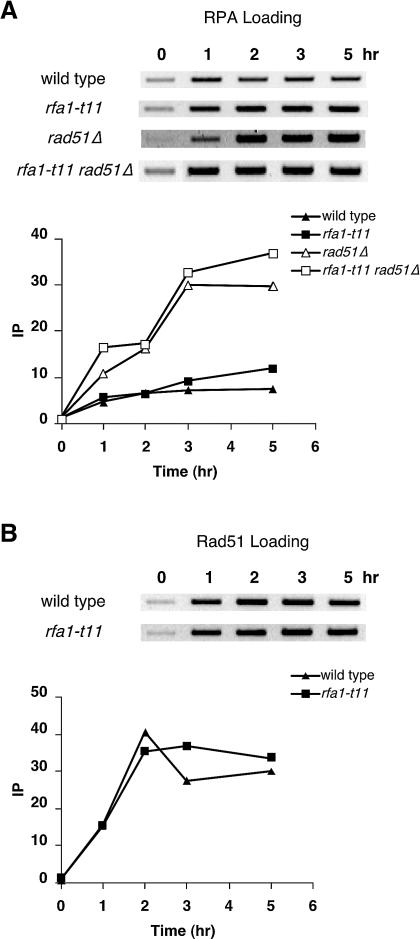
*rfa1-t11* Mutation Does Not Affect the Recruitment of Itself or Rad51 to an Unrepairable DSB (A) An unrepairable DSB was created in wild-type (yXW1), *rfa1-t11* (ySL31), *rad51*Δ (ySL306), and *rfa1-t11 rad51*Δ (ySL351) strains, and half of the DNA sample was immunoprecipitated with anti-Rfa1 antibody to extract *rfa1-t11*-bound chromatin. (B) For wild-type (yXW1) and *rfa1-t11* (ySL31) strains, the other half of the DNA sample was applied with anti-Rad51 antibody to extract Rad51-associated chromatin. PCR-amplified DNA from the *MAT* locus was run on ethidium bromide-stained gels (reverse images are shown). DNA signals were quantitated and graphed as described in Figure 2.

In a strain lacking *HML* and *HMR*, Rfa1-K45E binding was nearly identical to wild-type, both in a *RAD51* and in a *rad51*Γ background (Figure 5A). Moreover, binding of Rad51 to ssDNA at the HO-cut *MAT* locus was also comparable to that observed in wild-type cells (Figure 5B). Thus, the Rfa1-t11 protein is neither impaired in loading onto ssDNA, nor does it affect the loading of Rad51 in vivo.

**Figure 7:**
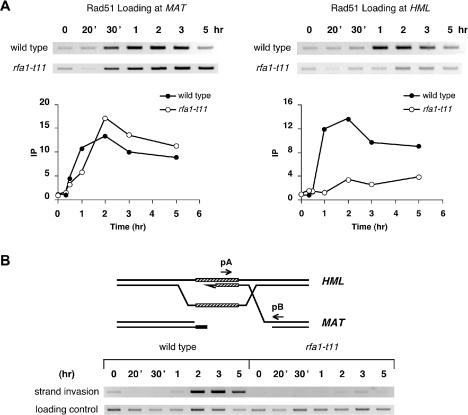
*rfa1-t11* Mutants Are Defective in the Strand Invasion Step of Gene Conversion (A) One half of the DNA extract collected from a typical timecourse experiment as described in Figure 6 was applied with anti-Rad51 antibody to immunoprecipitate Rad51-bound chromatin. Primers P1 and P2 and P1 and P3 were used to PCR-amplify Rad51-bound *MAT* and *HML* DNA, respectively (see Figure 4A). Samples were run on ethidium bromide-stained gels (reverse images are shown). DNA signals were quantitated and graphed as described in Figure 2. (B) Input DNA was used to PCR-amplify strand invasion product using a unique primer distal to *MAT* (pB) and a primer within the Yα sequence from *HML* (pA) (White and Haber 1990). PCR-amplified *ARG5,6* signals from the input DNA were used as loading control.

We then examined the effect of *rfa1-t11* during HO-induced switching of *MAT* **a** to *MAT*α, using *HML*α as the donor. As shown previously (Umezu et al. 1998), *rfa1-t11* strongly impaired *MAT* switching, with only 15% product evident after 5 h (Figure 6A). RPA bound normally to the *MAT* locus, but unlike what occurs in wild-type strains, its binding remained undiminished at later times (Figure 6B). Moreover, there was no increased association of RPA with *HML* over background levels (Figure 6B). When we examined Rad51 binding in this mutant, we found that Rad51 immunoprecipitated with *MAT* DNA, but not with *HML* (Figure 7A). In support of this important finding, we also used a PCR assay to show that *rfa1-t11* prevented the appearance of newly synthesized DNA using the 3′ end of the invading strand as a primer (Figure 7B). In this assay, a primer specific for the Yα region in *HML* (pA) can only amplify a strand invasion product with a primer specific for *MAT*-distal sequences (pB) if the 3′ end of the strand-invading DNA is extended by DNA polymerase at least 35 nucleotides (White and Haber 1990) (Figure 7B). These data strongly raise the possibility that RPA is required during the process of strand invasion and synapsis and not merely to facilitate formation of a Rad51 filament, as the binding of Rad51 to ssDNA at *MAT* seems to be normal in both kinetics and extent.

## Discussion

ChIP analysis provides a powerful tool for studying in vivo protein–DNA and protein–protein interactions. Using ChIP and related assays, we have demonstrated important roles of RPA during homologous recombination in vivo that could not have been known with certainty from in vitro studies. RPA is recruited to the DSB ends as soon as the DSB is detected on a Southern blot, and its binding precedes that of Rad51 (see Figures 2 and 4), which supports the idea that RPA is required to remove inhibitory secondary structures on ssDNA for Rad51 to polymerize across these regions (Sugiyama et al. 1997, 1998). This observation is also consistent with in vivo immunofluorescent staining results, suggesting that RPA foci appear earlier than Rad51 foci after irradiation (Golub et al. 1998; Gasior et al. 2001). Rad51 apparently displaces RPA from ssDNA, with the help of Rad52 (see Figure 3) and perhaps the Rad55/Rad57 auxiliary proteins. We note that our results are different from those reported by Wolner et al. (2003), who observed initial binding of RPA only after 45 min, whereas Rad51 was detected 25 min earlier, although it is not clear whether there is a statistically significant increase in Rad51 binding at the earliest time. In that assay, *RFA1* was tagged with 13 Myc epitope tags, which may have altered its behavior. We believe our results are consistent with the fact that RPA has a higher-affinity constant for ssDNA and is present in much greater abundance in the cell (Heyer and Kolodner 1989; Mazin et al. 2000; Sugawara et al. 2003).

We noticed that when RPA ChIP was carried out in donorless strains as well as in *rfa1-t11* strains that carry the donor loci, there was a continued presence of some RPA near the ends of a DSB. This may occur for several reasons. First, it is likely that the formation and maintenance of the Rad51 filament are a dynamic process, with subunits coming off the end and perhaps being replaced by RPA before being in turn replaced by Rad51. Second, the Rad51 nucleoprotein filament may not be, in vivo, a fully continuous structure, given that there are only about 3,500 monomers of Rad51 in the cell and that Rad51 binding is not highly cooperative (Mazin et al. 2000; Sugawara et al. 2003). Finally, the very ends of the DSB can religate and be recleaved by HO in a cycle that lasts several hours in the absence of donor sequences (and hence in the absence of homologous recombination) to repair the DSB (Frank-Vaillant and Marcand 2002). Thus, a fraction of molecules will be newly generated and will show RPA binding before Rad51, as we saw for the initial DSB.

The ChIP analysis presented here has shown that RPA is required for homologous recombination even after Rad51 has bound to ssDNA. First, RPA can immunoprecipitate donor sequences, the timing of which coincides with the loading of Rad51 at *HML* (see Figure 4). Second, the replication-proficient but recombination-deficient mutant of the largest subunit of RPA (*rfa1-t11*) is able to allow Rad51 to bind to ssDNA, but is incapable of forming normal levels of strand invasion and primer extension products (see Figures 6 and 7). We offer two possible explanations for this unexpected finding. First, whereas Rad51 can bind to ssDNA in *rfa1-t11*, it may not establish a functional filament capable of carrying out a search for homology and strand invasion, even though the association of Rad51 with ssDNA appears to be normal. But the defect in cells with *rfa1-t11* seems different from that seen in cells lacking Rad55 (Sugawara et al. 2003), where there was delayed and less-extensive binding of Rad51 to ssDNA; moreover, although Rad51 eventually bound, it was unable to catalyze synapsis between *MAT* ssDNA and *HML*. In *rad55*Γ cells, it is likely that the Rad51 filament is discontinuous and unable to function. However, with *rfa1-t11*, the loading of Rad51 onto ssDNA appears to be identical to that seen in wild-type cells (see Figures 5B and 7A).

Alternatively, in *rfa1-t11* cells, the filament may indeed be functional, but RPA is needed to stabilize the strand invasion intermediate and *rfa1-t11* is unable to carry this out. RPA may be required to bind to the displaced D-loop, to prevent rapid reversal of the process, which is implicated by in vitro studies of strand exchange (Eggler et al. 2002). In that study, extensive heteroduplex could be formed without RPA, as revealed by psoralen cross-linking of joint molecule DNA before removal of Rad51 by deproteinization, but without cross-linking, the deproteinized joint molecule DNA fell apart into the original single-stranded and double-stranded substrates very quickly. An analogous role for SSB has been suggested in RecA-mediated strand invasion (Lavery and Kowalczykowski 1992), in which SSB prevents the reversal of DNA strand exchange by removing the displaced single strand. It is possible that the Rfa1-K45E mutation renders the mutant RPA complex unable to bind to the displaced ssDNA at *HML* and thus unable to carry out strand exchange, while binding to *MAT* ssDNA that has a free 3′ end tail is not affected.

Both in vitro (Kantake et al. 2003) and in vivo analyses showed that *rfa1-t11* was able to bind to ssDNA very similarly to wild-type, but our in vivo data did not see any significant impairment of its Rad52-mediated displacement by Rad51. It should be noted that the inhibition of Rad51-mediated strand exchange by *rfa1-t11* in vitro was carried out with saturating amounts of Rad51 (whereas the amount of Rad51 in the cell is quite limited) and that the inhibition of Rad51-mediated strand exchange was impaired primarily when RPA was present in excess (Kantake et al. 2003). How these conditions relate to those prevailing in vivo remains unknown. In this regard, it is also noteworthy that in vitro studies did not see any impairment of single-strand annealing (Kantake et al. 2003), whereas in vivo, single-strand annealing is nearly eliminated in *rfa1-t11* strains (Umezu et al. 1998). Further comparisons of in vitro and in vivo data will be valuable in understanding how the more complex environment within the cell affects processes of recombination.

## Materials and Methods

### 

#### Strains

Donorless strains are isogenic derivatives of JKM139, which has the genotype of *ho*Δ *hml*Δ::*ADE1 MAT* **a** *hmr*Δ::*ADE1 ura3–52 leu2–3,112 trp1*::*hisG lys5 ade1–100 ade3*::*GAL*::*HO*. The wild-type strain yXW1 was constructed by transforming JKM139 with pGI4 (*bar1*::*ADE3*) (Wach et al. 1994). ySL83 contains *yku80*Δ::*KAN* and *bar1*::*TRP1*. ySL306 and ySL177 contain *rad51*Δ::*URA3* and *rad52*Δ::*TRP1*, respectively (Lee et al. 1998, 2001). ySL31 has the point mutation (K45E) in the largest subunit of RPA (Lee et al. 1998), and ySL351 was derived from ySL31 and contains *rad51*Δ::*LEU2.* Strains capable of undergoing DSB-induced gene conversion were derived from OAy470 (Aparicio et al. 1997), which has the genotype of *ho*Δ *MAT* **a** *ura3–1 trp1–1 leu2–3,112 his3–11,15 ade2–1 can1–100 bar1*::*hisG*. A galactose-inducible *GAL*::*HO* gene was integrated at *ADE3* of OAy470 using YIPade3HO constructed by L. L. Sandell (Sandell and Zakian 1993) to obtain the wild-type strain yXW2. yXW3 is an isogenic derivative of yXW2, into which the point mutation (K45E) of Rfa1 was introduced by integration and excision of a YIp5 (*URA3*-containing) plasmid (Lee et al. 1998).

#### DNA analysis

When cells were harvested for ChIP at intervals after induction of HO (see below), a second set of DNA samples were collected for Southern blot analysis as described before (White and Haber 1990). The strand invasion/primer extension assay in Figure 7B was previously described (White and Haber 1990). The primers used were 5′-GCAGCACGGAATATGGGACT-3′ (pA) and 5′-ATGTGAACCGCATGGGCAGT-3′ (pB).

#### ChIP

ChIP was carried out as described previously with minor modifications (Dedon et al. 1991; Sugawara et al. 2003). Cells were pregrown to a density between 5 × 10^6^ and 1 × 10^7^ cells/ml in YP–lactate medium and HO endonuclease was induced by addition of 2% galactose. Strains undergoing DSB-induced gene conversion were treated with 2% glucose after 1 h to repress further cutting by HO. Proteins were cross-linked by addition of 1% (final concentration) formaldehyde to 45 ml of culture for 10 min, followed by quenching with 125 mM glycine (final concentration) for 5 min. Cells were lysed with glass beads, and the extracts were sonicated to shear the DNA to an average size of 0.5 kb. Extracts were then divided into IP and input samples (12:1 ratio). IP samples were split. Half of the extract was incubated with polyclonal anti-Rfa1 antibody (kindly provided by S. Brill) for 1 h at 4°C and bound to protein G–agarose beads for 1 h at 4°C. In the ChIP experiments described in Figure 2, Figure 4D, Figure 5B, Figure 6, and Figure 7, the other half of the extract was incubated with affinity-purified anti-Rad51 antibody (provided by P. Sung) or unpurified antibody (provided by A. Shinohara) for 1 h at 4°C and bound to protein A–agarose beads for 1 h at 4°C. The protein-bound beads were carried through a series of washes, followed by elution of the proteins and reversal of cross-linking (6 h at 65°C). Samples were treated with proteinase K followed by phenol extraction and ethanol precipitation.

In the control experiments described in Figure 1D, 4 ng of purified single-stranded β-lactamase (*AMP*) gene DNA from plasmid pBR322 was added at the time of cell breakage. IP and input samples were further subject to PCR to test the presence of the *AMP* sequences.

#### PCR amplification

The *MAT*-specific primers were 5′-TCCCCATCGTCTTGCTCT-3′ (P1) and 5′-GCATGGGCAGTTTACCTTTAC-3′ (P2), which amplifies a PCR product of 293 bp. The *HML*-specific primers were 5′-TCCCCATCGTCTTGCTCT-3′ (P1) and 5′-CCCAAGGCTTAGTATACACATCC-3′ (P3), which amplifies a PCR product of 280 bp. Primers used for the amplification of the sites proximal to the DSB (see Figure 1C) were −29.8 kb, 5′-TCGTCGTCGCCATCATTTTC-3′ and 5′-GCCCAAGTTTGAGAGAGGTTGC-3′; −16.6 kb, 5′-CGTCTTCTCAGCGAACAACAGC-3′ and 5′-GCAATAACCCACGGAAACACTG-3′; −9.5 kb, 5′-TCAGGGTCTGGTGGAAGGAATG-3′ and 5′-CAAAGGTGGCAGTTGTTGAACC-3′; −5.3 kb, 5′-ATTGCGACAAGGCTTCACCC-3′ and 5′-CACATCACAGGTTTATTGGTTCCC-3′; −3.6 kb, 5′-ATTCTGCCATTCAGGGACAGCG-3′ and 5′-CGTGGGAAAAGTAATCCGATGC-3′; −1.6 kb, 5′-ATGTCCTGACTTCTTTTGACGAGG-3′ and 5′-ACGACCTATTTGTAACCGCACG-3′; and −0.2 kb, 5′-AAAGAAGAAGTTGCAAAGAAATGTGG-3′ and 5′-TGTTGCGGAAAGCTGAAACTAAAAG-3′. Oligos used for the sites distal to the DSB were 0.2 kb, 5′-CCTGGTTTTGGTTTTGTAGAGTGG-3′ and 5′-GAGCAAGACGATGGGGAGTTTC-3′; 2.1 kb, 5′-GCCTCTATGTCCCCATCTTGTCTC-3′ and 5′-GTGTTCCCGATTCAGTTTGACG-3′; 3.1 kb, 5′-TAACCAGCAATACCAAGACAGCAC-3′ and 5′-TTTTACCTACCGCACCTTCTAAGC-3′; 5.7 kb, 5′-CCAAGGAACTAATGATCTAAGCACA-3′ and 5′-ACCAGCAGTAATAAGTCGTCCTGA-3′; and 9.5 kb, 5′-TGGATCATGGACAAGGTCCTAC-3′ and 5′-GGCGAAAACAATGGCACTCT-3′.

These PCR primers gave products of about 300 bp. Primers specific for the *ARG5,6* locus were either 5′-AGAAAGGGGGTATTATCAATGGCTC-3′ and 5′-AGGAAAATCACGGCGCAAAA-3′, which amplifies a PCR product of 533 bp, or 5′-CAAGGATCCAGCAAAGTTGGGTGAAGTATGGTA-3′ and 5′-GAAGGATCCAAATTTGTCTAGTGTGGGAACG-3′, which amplifies a PCR product of 381 bp. Normalization using these two different pairs of primers has been shown not to affect the final quantification results. Primers used for the amplification of the *AMP* sequences (see Figure 1D) were 5′-GAAGACGAAAGGGCCTCGTG-3′ and 5′-GCTGCAGGCATCGTGGTGTC-3′, which amplifies a PCR product of 750 bp. All PCR assays were accompanied by reactions using dilutions of the 0-h input sample to assess the linearity of the PCR signal and to create a calibration curve, as described before (Sugawara et al. 2003). Samples were run on ethidium bromide-stained agarose gels (1.4%) and quantitated using an Innotech Alphaimager™ and Quantity One software™ (BioRad, Hercules, California, United States), which was also used to correct for minor deviations from a linear response in signal. Quantification and graphing were carried out as described previously with minor changes (Sugawara et al. 2003). For RPA ChIP analysis, all IP samples were first normalized to IP signals from an independent locus (*ARG5,6*) on chromosome V in a multiplex experiment, by using *ARG5,6* and *MAT* or *HML* primers in the same PCRs. This was accomplished by dividing each *MAT* or *HML* IP signal by the corresponding *ARG5,6* IP signal to correct for differing amounts of chromatin collected at each timepoint. Then *MAT* or *HML* IP signals at later timepoints were normalized and graphed to the 0-h IP signal to measure the relative increase. For Rad51 ChIP analysis, quantification and graphing were carried out as described before (Sugawara et al. 2003), in which all IP samples were normalized to the *ARG5,6* input signals at the respective time points. Graphing represents the average of at least three independent ChIP timecourse experiments for each strain.

## Supporting Information

### Accession Numbers

The *Saccharomyces* Genome Database (http://www.yeastgenome.org/) ID accession numbers for the entities discussed in this paper are *ARG5,6* (S0000871), *HML* (L0000791), *HMR* (L0000792), *MAT* (L0001031), Rad51 (S0000897), Rad52 (S0004494), Rad55 (S0002483), Rad57 (S0002411), *RFA1* (S0000065), *RFA2* (S0005256), and *RFA3* (S0003709).

## Abbreviations

ChIP: chromatin immunoprecipitation
DSB: double-strand break
dsDNA: double-stranded DNA
IP: immunoprecipitation
*MAT*: mating type
RPA: replication protein A
SSB: single-stranded binding protein
ssDNA: single-stranded DNA
